# Glucocorticoid Regulation of Astrocytic Fate and Function

**DOI:** 10.1371/journal.pone.0022419

**Published:** 2011-07-21

**Authors:** Shuang Yu, Silei Yang, Florian Holsboer, Nuno Sousa, Osborne F. X. Almeida

**Affiliations:** 1 Max Planck Institute of Psychiatry, Munich, Germany; 2 Life and Health Sciences Research Institute (ICVS), School of Health Sciences, University of Minho, Braga, Portugal; University of South Florida, United States of America

## Abstract

Glial loss in the hippocampus has been suggested as a factor in the pathogenesis of stress-related brain disorders that are characterized by dysregulated glucocorticoid (GC) secretion. However, little is known about the regulation of astrocytic fate by GC. Here, we show that astrocytes derived from the rat hippocampus undergo growth inhibition and display moderate activation of caspase 3 after exposure to GC. Importantly, the latter event, observed both *in situ* and in primary astrocytic cultures is not followed by either early- or late-stage apoptosis, as monitored by stage I or stage II DNA fragmentation. Thus, unlike hippocampal granule neurons, astrocytes are resistant to GC-induced apoptosis; this resistance is due to lower production of reactive oxygen species (ROS) and a greater buffering capacity against the cytotoxic actions of ROS. We also show that GC influence hippocampal cell fate by inducing the expression of astrocyte-derived growth factors implicated in the control of neural precursor cell proliferation. Together, our results suggest that GC instigate a hitherto unknown dialog between astrocytes and neural progenitors, adding a new facet to understanding how GC influence the cytoarchitecture of the hippocampus.

## Introduction

Stress and glucocorticoid (GC) hypersecretion during antenatal, neonatal, adolescent and adult life are implicated in a number of brain disorders, including major depression [1], [2], dementia [3], addiction [4] and schizophrenia [5], [6]. Neuroimaging studies in humans reveal a strong negative correlation between cortisol levels and hippocampal volume in patients with major depression [7], [8]; importantly, there is a positive association between cognitive function and cortisol levels [7], [9]. Similar structure-behavior relationships have been reported in the hippocampus, and other brain regions, of laboratory rodents experiencing high GC levels [10]–[14]. These volumetric changes have been ascribed to neuronal atrophy [15] and glial cell loss [16]. Supporting the latter, postmortem studies report reduced glial densities and numbers in the prefrontal cortex (PFC) [17], amygdala [18] and hippocampus [16] of depressed patients, and Banasr and Duman [19] demonstrated that chemical ablation of astrocytes in the PFC results in depressive-like behavior in rats. Moreover, chronic stress has been shown to induce astrocytic loss in the hippocampus [20], an effect that can be reversed by drugs with antidepressant actions. While Banasr et al. [21] found that chronic stress interferes with glial cell metabolism, through glutamatergic mechanisms, it remains unclear as to whether GC are causally involved in the loss of astrocytes after stress. The present study provides unequivocal evidence that astrocytes respond to GC with growth inhibition rather than apoptosis. Moreover, this study shows, for the first time, that GC modify astrocytic production of various growth factors that ultimately inhibit the proliferation of neural precursors in the hippocampus.

## Results

### Astrocytes escape GC-induced apoptosis during development and adulthood

**Figure 1:**
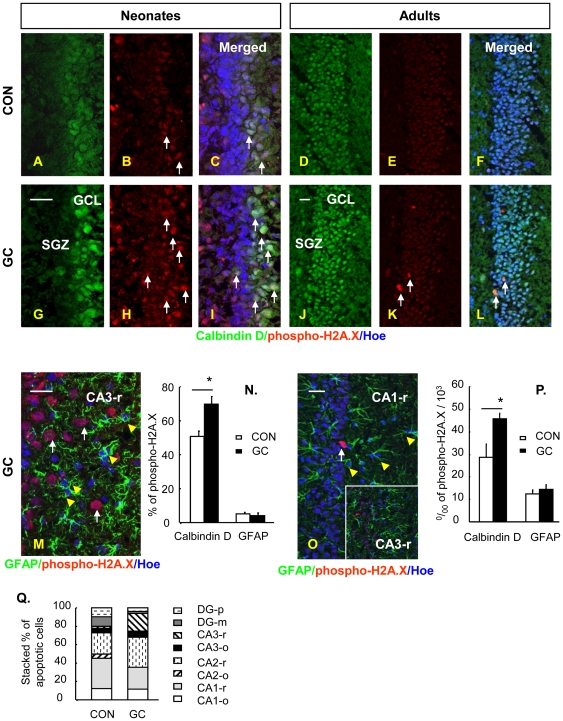
GC treatment drives neurons but not astrocytes into apoptosis in neonatal and adult rats. **A**–**L**, Representative confocal images showing double staining for calbindin-D28K (**A**, **D**, **G**, **J**) and phosho-H2A.X (**B**, **E**, **H**, **K**) in the dentate gyrus of control and GC-treated neonatal (**A**–**C, G**–**I**) and adult (**D**–**F, J**–**L**) rats. Hoechst 33342 staining was used to identify cell nuclei and to help delineate the SGZ and GCL. Arrows indicate the representative positive phosphor-H2A.X staining in calbindin-D28K positive neurons. **M** and **O**, are representative images showing double-staining of GFAP and phospho-H2A.X in the *stratum radiatum* of the hippocampal CA3 and CA1 subfields (CA3-r, CA1-r) in GC-treated neonatal (**M**) and adult (**O**) rats. Arrowheads indicate GFAP-positive astrocytes that were negative for phospho-H2A.X, an early marker of apoptosis. Arrows indicate the representative phosphor-H2A.X staining in GFAP-negative cells. **N** and **P** illustrate the significant increase of apoptosis in calbindin-positive neurons, but not GFAP-labeled astrocytes, in neonatal (**N**) and adult (**P**) rats treated with GC (dexamethasone, 200 µg/kg/d on days 1–3, tapering to 100 µg/kg/d on days 4–7). The counts are from all hippocampal subregions displaying positive signal for calbindin (granule cell layer of DG) or GFAP (molecular and polymorphic cell layers of DG, and the *strata oriens* and *radiatum* of CA1-CA3). **Q**, Stacking figure showing that GC treatment does not induce apoptosis in astrocytes in any hippocampal subfield, as indicated by double-staining of GFAP and phopho-H2A.X. The relative numbers (%) of phospho-H2A.X^+^/GFAP^+^ cells relative to total GFAP^+^ cells in each subfield were calculated; each value was used to create the stacking figure in which each column represents the % of apoptotic events in astrocytes in each subfield *vs.* the total number of apoptotic events in astrocytes in the whole hippocampal formation (100%). o, *stratum oriens*; m, molecular layer; p, polymorphic cell layer. r, *stratum radiatum*. * p<0.05 compared to CON. Scale bars: 20 µm.

It has been reported that hippocampal astrocyte numbers are reduced in GC-related disorders [16], [20], suggesting that GC have a detrimental effect on astrocyte generation or survival. In the present study we monitored the *in situ* expression of phospho-H2A.X, a marker of early apoptosis, in GFAP-positive cells (astrocytes) within the hippocampal formation of GC-treated neonatal (1 day old) and adult (3-month old) rats. Results demonstrate very low co-localization of phospho-H2A.X and GFAP immunoreactivity in the hippocampus (all subfields) of neonatal (∼5%) and adult (∼1%) rats (Fig. 1M-Q), indicating refractoriness of astrocytes to GC-triggered apoptosis. These findings contrast strikingly with those previously reported by us with respect to neural precursor cells [22] and mature neurons [23]–[26], and replicated in this study: specifically, we here show that a significant number of calbindin D28K-positive cells (neurons) express phospho-H2A.X upon exposure to GC, an effect that was evident in both, the neonatal and adult hippocampus (Fig. 1A–L, N, P).

### Phenotypic identity of GC-sensitive hippocampal cells and mechanisms contributing to GC-insensitivity

**Figure 2:**
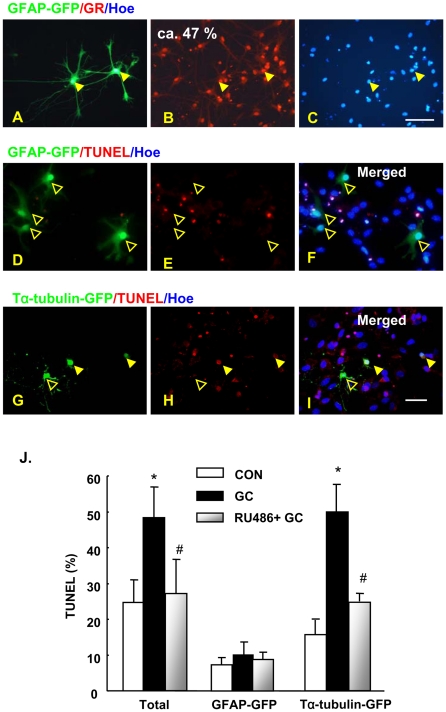
Astrocytes are spared from GC-triggered apoptosis in primary hippocampal cultures. Hippocampal cultures were genetically tagged with either Tα-tubulin-GFP or GFAP-GFP plasmids, to identify neurons and astrocytes, respectively. Approximately 50% of astrocytes in typical cultures displayed GR immunoreactivity (**A–C**, examples shown by arrowheads). After exposure to GC or vehicle, apoptosis in the different cell populations was visualized by TUNEL and Hoechst 33342 histochemistry (**D–F and G–I**). Solid arrowheads exemplify GFP^+^ cells that entered apoptosis after GC treatment; open arrowheads indicate non-apoptotic GFP-transfected cells. Numerical data (mean ± SD) from analysis of TUNEL staining in either all cells in culture, Tα-tubulin-GFP or GFAP-GFAP sub-populations are depicted in (**J**). * p<0.05 vs. CON, # p<0.05 vs. DEX. Scale bars: 50 µm in **A–C** and 20 µm in **D–I**.

**Figure 3:**
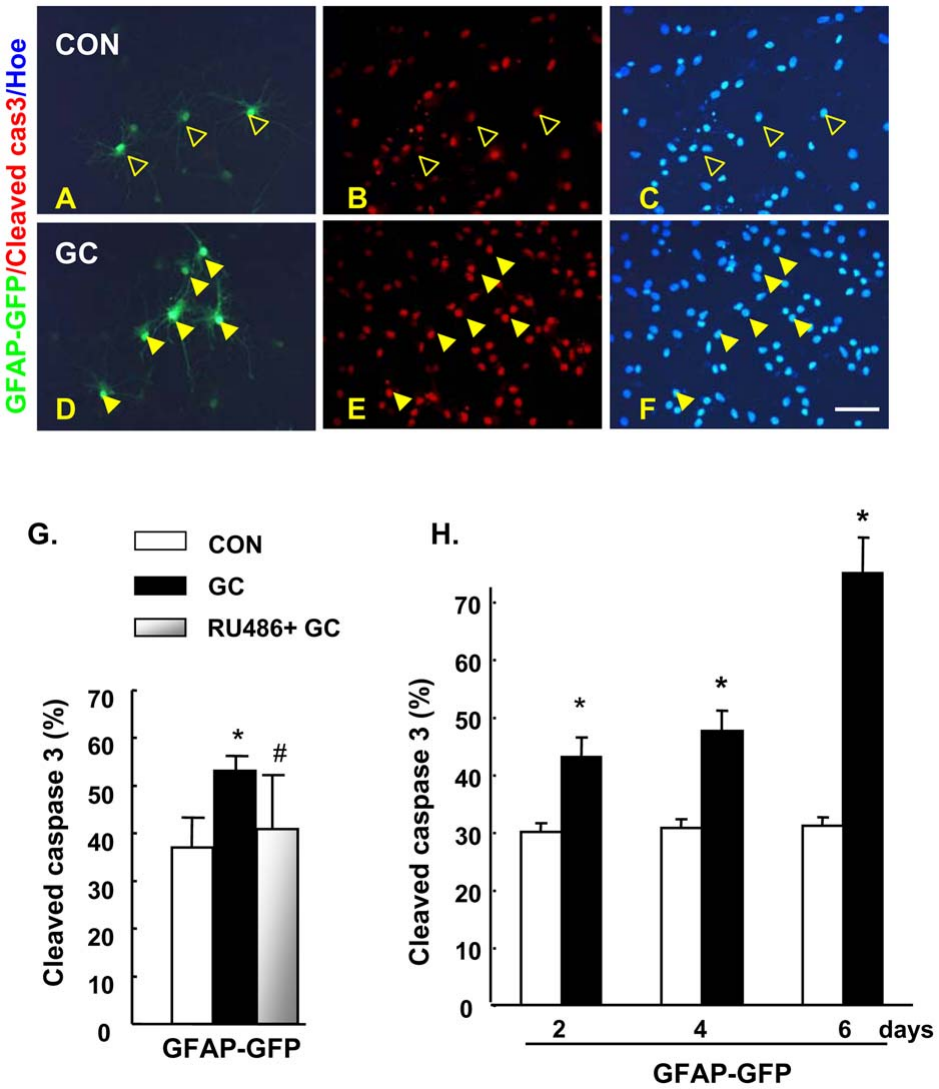
Caspase 3 is activated by GC-treated in astrocytes grown in mixed hippocampal cultures. As compared to vehicle-treated GFAP-GFP-labeled astrocytes (**A–C**), those treated with GC (**D–F**) displayed moderate levels of activated caspase 3. Open arrowheads indicate GFAP-GFP^+^/cleaved caspase 3^−^ staining; solid arrowheads indicate GFAP-GFP+/cleaved caspase 3^+^ cells. The GR antagonist RU38486 significantly attenuated GC-stimulated activation of caspase 3 (**G**). Extended exposure of cultures to GC (48–144 h) led to a progressive increase in activated caspase 3 immunoreactivity in GFAP-GFP tagged astrocytes (**H**), without causing significant apoptosis monitored by TUNEL and Hoechst staining (not shown). All numerical data represent mean ± SD. * p<0.05 vs. CON, # p<0.05 vs. GC. Scale bar: 50 µm.

The incidence of GC-triggered apoptosis was monitored by TUNEL and Hoechst 33342 histochemistry in mixed hippocampal cultures transfected with GFP-driven neuron- (Tα1-GFP) [27], [28] and astrocyte- (GFAP-GFP) [29] specific plasmids; the genetic tagging approach excluded the possibility that astrocytes undergoing apoptosis might have lost their GFAP antigenicity. As shown previously [26], maximum apoptotic effects were seen in the primary hippocampal cultures when DEX was applied at 10^−5^ M, a dose used in all subsequent experiments. This analysis revealed that neurons (Fig. 2G-I and J), but not astrocytes (Fig. 2D–F and J), are sensitive to the apoptotic actions of GC. While confirming results reported in the previous section, this experiment also revealed that GC treatment increases the expression of active (cleaved) caspase 3 in astrocytes (Fig. 3A–F); in fact, extended exposure to GC (up to 144 h) was accompanied by further increases of activated caspase 3 levels (Fig. 3H) but, nevertheless, without any significant increase of apoptotic events (data not shown).

The neuronal effects of GC were prevented by pre-application of mifepristone (RU38486; 10^−5^ M), a glucocorticoid receptor (GR) antagonist, indicating their mediation through GR (Fig. 2J). In astrocytes, which also express GR (Fig. 2A–C), mifepristone abolished the ability of GC to stimulate active caspase 3 levels (Fig. 3A–G). Thus, the resilience of astrocytes to the apoptotic actions of GC most likely reflects the intrinsically different cellular machineries in astrocytes and neurons.

**Figure 4:**
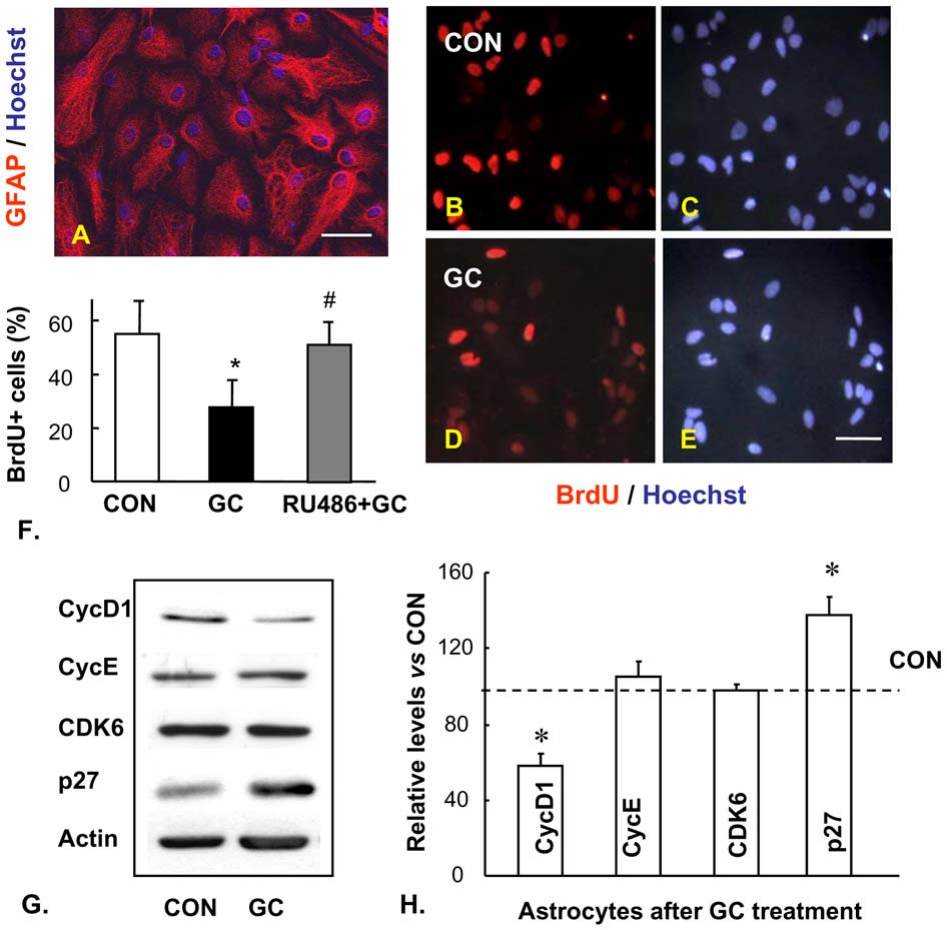
Anti-proliferative actions of GC in astrocytes are GR-dependent. **A,** Representative immunostaining of GFAP in the enriched astrocytic culture. **B–E**, are representative images showing BrdU incorporation in control (**B, C**) and GC-treated (dexamethasone, at 10^−5^ M for 48 h in medium with charcoal-stripped serum) astrocytes (**D, E**). Hoechst 33342 counterstaining demonstrates comparable cell densities. BrdU (20 µM) was added to cultures 12 h before fixation. **F,** shows that the anti-proliferative actions of GC are counteracted by addition of the GR antagonist, RU38486 (10^−5^ M). **G**, Representative Western blots showing GC (dexamethasone; 10^−5^ M in medium supplemented with charcoal-stripped serum; 48 h) regulation of various key regulators of the cell cycle in cultured astrocytes; the semi-quantitative (n = 4) data from these immunoblotting experiments are shown in **H.** Note that while GC treatment downregulates cyclin D1 protein expression, the treatment results in a concomitant increase in the levels of the cell cycle inhibitor, p27. Cyclin E and CDK6 expression levels are not changed after exposing astrocytes to GC. Numerical data represent mean ± SD. * p<0.05 vs. CON, # p<0.05 vs. GC. Scale bar: 50 µm.

Further studies were carried out in astrocyte-enriched (>90%, Fig. 4A) cultures to examine the intrinsic responses of astrocytes to GC, to specifically exclude potential confounds resulting from their juxtaposition to neurons in the mixed-cell cultures. Extending our previous demonstration that GC inhibit proliferation of neural cells in culture [30], we now show that enriched astrocyte cultures also exhibit growth inhibition upon exposure to GC and that the GC effect is abrogated in the presence of the GR antagonist, mifespristone (Fig. 4B–F). Fluctuations in the levels of cyclins and cyclin-dependent kinases (CDK), as well as cell cycle inhibitors, determine the progress of the cell cycle and proliferative capacity [31]. Here, we show by immunoblotting that GC respectively down- and upregulate the expression of cyclin D1 and the cell cycle inhibitor p27 in astrocytes (Fig. 4G and H). These effects appear to be selective insofar that the levels of other cyclins (e.g. cyclin E) and CDK6 were not significantly influenced by GC treatment (Fig. 4G and H).

**Figure 5:**
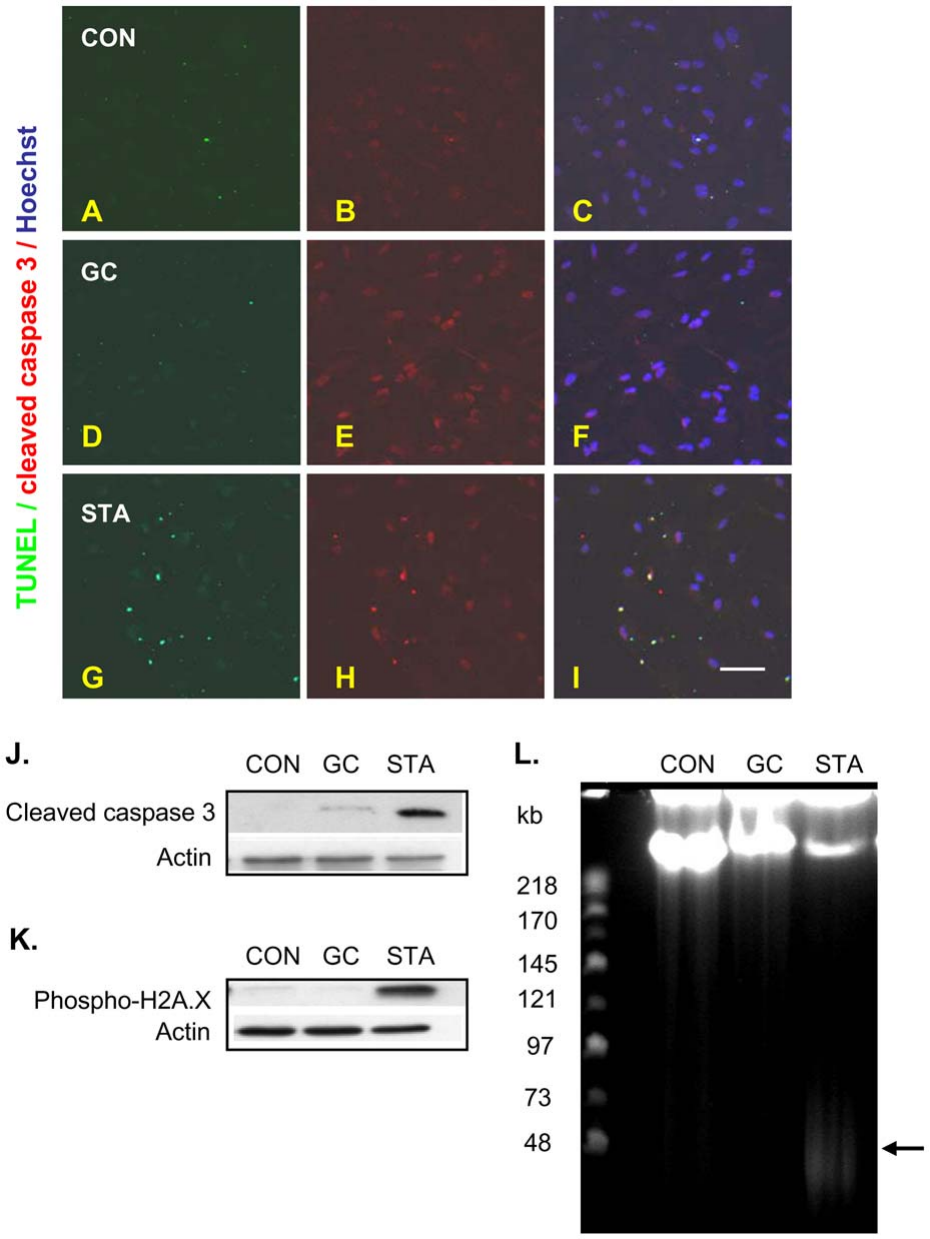
Enriched astrocytic cultures also respond to GC with moderate activation of caspase 3, but fail to show signs of early- or late-stage apoptosis. After re-plating, enriched astrocytic cultures were treated with GC (48 h) in medium containing either charcoal-stripped serum (data not shown) or B27 supplement (representative images in **A–L**). Enriched astrocytes responded to GC treatment DEX with moderately increased immunostaining for activated caspase 3; these cells did not enter late-stage (stage II) apoptosis, as shown by TUNEL (**A–F**). In contrast, staurosporine (STA) induced a marked activation of caspase 3 and apoptosis (**G–I**). The immunocytochemical results shown for activated caspase 3 in **A–I** were confirmed by immunoblotting (J). Staurosporine, but not GC, treatment of enriched astrocytic cultures significantly increased levels of immunoreactive phospho-H2A.X, a marker of early apoptosis, as shown by immunoblotting studies (**K**). Similarly, astrocytes exposed to STA, but not GC, displayed high molecular weight (HMW) DNA fragments, when lysates where subjected to pulse-field gel electrophoresis (PFGE) (**L**); all lanes were loaded with DNA from the same number of astrocytes, and arrow indicates 50 kb HMW DNA fragments. Scale bars: 50 µm.

**Figure 6:**
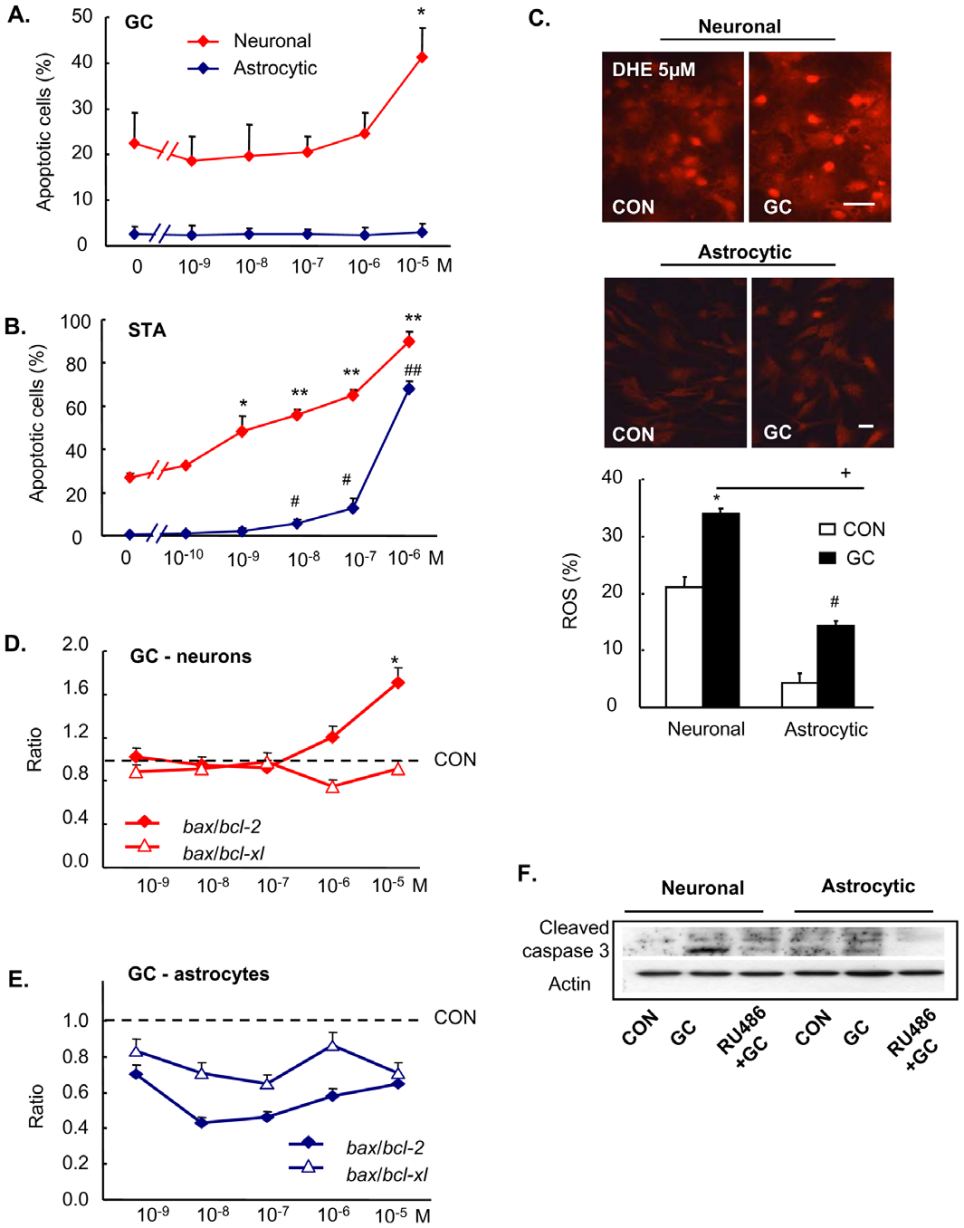
Insights into potential mechanisms underlying astrocytic resistance to GC-induced apoptosis. Astrocytes show less susceptibility to GC- (**A**) and staurosporine (STA)-induced (**B**) apoptosis, as compared to neuronal cells. Both neurons and astrocytes respond to GC treatment with a significant increase of ROS (measured by fluorescent DHE nuclear translocation) (**C**); note that, as compared to neurons, astrocytes generate markedly lower levels of ROS under basal conditions and after GC treatment. The ratios of expression of mRNAs for pro- vs. anti-apoptotic members of the Bcl2 family (*bax* vs. *bcl-X_L_* and *bcl-2*) are different in neurons and astrocytes (**D and E**); mRNA levels were determined by qPCR. Neurons and astrocytes also respond differentially to GC treatment in terms of their activated caspase 3 responses (measured by immunoblotting) (**F**), with astrocytes showing smaller increases in levels of activated caspase 3. Numerical data are shown as mean ± SD. * p<0.05 vs. neuron CON; # p<0.05 vs. astrocyte CON; + p<0.05 GC-treated neurons vs. GC-treated astrocytes. Scale bars: 25 µm.

Astrocytes grown in either serum-free, chemically-defined medium (Neurobasal A/B27, also used for the mixed-cell cultures) or standard medium (DMEM), supplemented with charcoal-stripped (steroid-free) serum, displayed moderate increases in immunoreactive caspase 3 (active form), but failed to show signs of apoptosis upon treatment with GC (10^−9^–10^−5^ M), as revealed by TUNEL and active caspase 3 histochemistry (Fig. 5A–F, J; also see Fig. 6A). On the other hand, the astrocytic cultures showed significant levels of caspase 3 activation and apoptosis when treated with staurosporine (50 nM), a protein kinase inhibitor and general apoptotic agent (Fig. 5G–I, J). Notably, the dose-response curves showing astrocytic vs. neuronal apoptotic responses to staurosporine reveal that astrocytes are less vulnerable to apoptosis (Fig. 6B).

Apoptotic DNA fragmentation is a two-stage process in which the DNA is first cleaved into large fragments of 50–300 kb (high molecular weight [HMW] DNA fragmentation), followed by subsequent inter-nucleosomal cleavage into low molecular weight (LMW) fragments [32]. Although LMW fragmentation (identified by TUNEL, DNA laddering, Hoechst staining) is a widely used marker of apoptosis, there is strong evidence that apoptosis in certain cells, and under specific conditions, may be marked by HMW DNA fragmentation [33]. Accordingly, extracts from GC-treated astrocytes were subjected to pulse-field gel electrophoresis and immunoblotting with an antibody against phospho-H2A.X which marks one of the earliest cellular responses to DNA damage that subsequently leads to apoptosis [34]. As shown in Figs. 5L and K, neither HMW DNA fragmentation nor phospho-H2A.X levels were increased when astrocytes were exposed to GC. In contrast, both markers were strongly evident in extracts from staurosporine-treated astrocytes (Fig. 5L and K).

Together, the findings reported thus far in this section suggest that the differential GC-induced apoptotic response of astrocytes and neurons reflects divergent post-receptor cellular responses by the two cell types. At the same time, the results indicate that, as compared to neurons, astrocytes are endowed with mechanisms that allow them to more effectively buffer the actions of apoptotic stimuli.

Since mitochondria play a critical role in the regulation of apoptosis, including GC-induced apoptosis [35], our initial investigations into factors and mechanisms that could potentially render astrocytes resistant to GC-induced apoptosis focused on mitochondrial function. Previous studies have shown that GC increase neuronal ROS levels [36], [37]. High levels of cellular ROS, generated by mitochondria as by-products of cellular metabolism, result in oxidative damage of DNA and other macromolecules and ultimately lead to cell senescence and death [38]. Here, we asked whether differences in the rates of ROS generation by neurons and astrocytes can explain their differential sensitivity to GC-induced apoptosis. By monitoring ethidium intercalation into DNA, we found that, as compared to astrocytes, neurons produce significantly higher levels of ROS under basal conditions, as well as after GC treatment (Fig. 6C).

The mitochondrial or intrinsic pathway of apoptosis is rheostatically controlled by pro- and anti-apoptotic proteins [38] and we previously showed that GC-induced apoptosis in hippocampal neurons is determined by the relative expression levels of pro- (Bax) and anti- (BCl-xl, BCl-2) apoptotic molecules [24]. Results depicted in Fig. 6D and E show that whereas GC dose-dependently increases the ratio of *bax*:*bcl-2* mRNA levels (*bax:bcl-xl* ratios were unchanged) in neurons, astrocytes do not exhibit major alterations in these profiles; these expression profiles correlated with the extent of activation of caspase 3 (high in neurons that ultimately underwent apoptosis, low in astrocytes which resisted apoptosis; Fig. 6F). These findings suggest that differences in the ability of astrocytes and neurons to buffer the cellular actions of GC contribute to their differential vulnerability to GC-induced apoptosis.

### GC regulation of astrocytic cytokines and neuronal cell turnover

**Figure 7:**
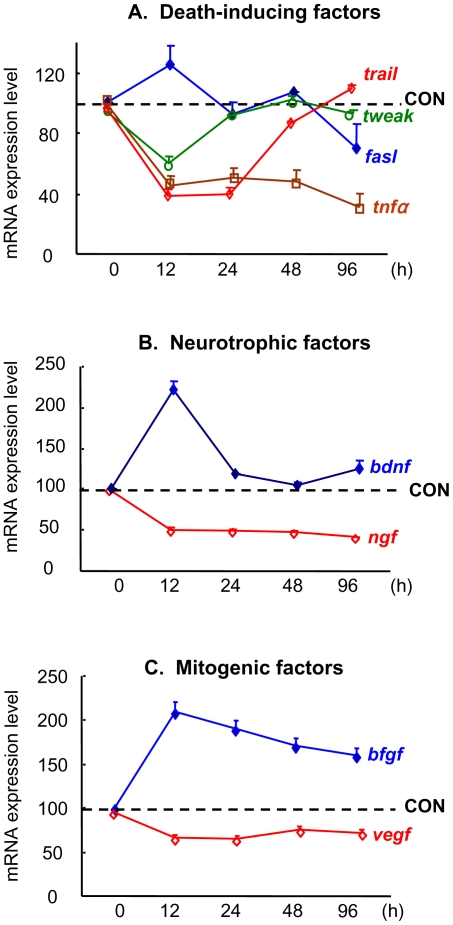
Temporal mRNA expression profiles of growth- and survival-regulating peptides in GC-treated astrocytes. **A,** molecules implicated in the extrinsic death pathway; FasL mRNA was transiently increased, TRAIL and TWEAK mRNAs showed transient reductions and TNFα mRNA showed a sustained reduction after application of GC. **B**, expression patterns of the mRNAs encoding the neurotrophic factors BDNF (transient upregulation) and NGF (sustained downregulation) after GC treatment. **C**, levels of mRNA encoding for the mitogenic factors bFGF (increased) and VEGF (decreased) following exposure of astrocytes to GC. In all analyses, *gapdh* and *actin* served as housing-keeping gene controls. Values shown derive from 3 independent experiments (mean ± SD).

Astroyctes produce a large number of soluble, membrane-bound proteins and peptides under basal conditions and in response to neuronal insults. Whereas anisomorphic or reactive astrogliosis leads to exacerbation of the effects of insults, astrocyte activation (or isomorphic astrogliosis) is thought to play a role in promoting neuronal survival, repair and proliferation [39]. Since the expression of several astrocyte-derived cytokines is known to be regulated by GC [40], [41], we here focused on those implicated in neurogenesis and neuronal survival. Analysis by qPCR revealed that GC regulate the expression of a number of cytokine genes whose products could potentially influence the fate of neurons through paracrine mechanisms. Specifically, GC significantly altered the expression of mRNAs encoding death-inducing factors (*fasL, trail, tweak* and *tnfα,* Fig. 7A), neurotrophic factors (*bdnf, ngf,* Fig. 7B) and mitogenic factors (*bfgf, vegf,* Fig. 7C) in astrocyte-enriched cultures.

**Figure 8:**
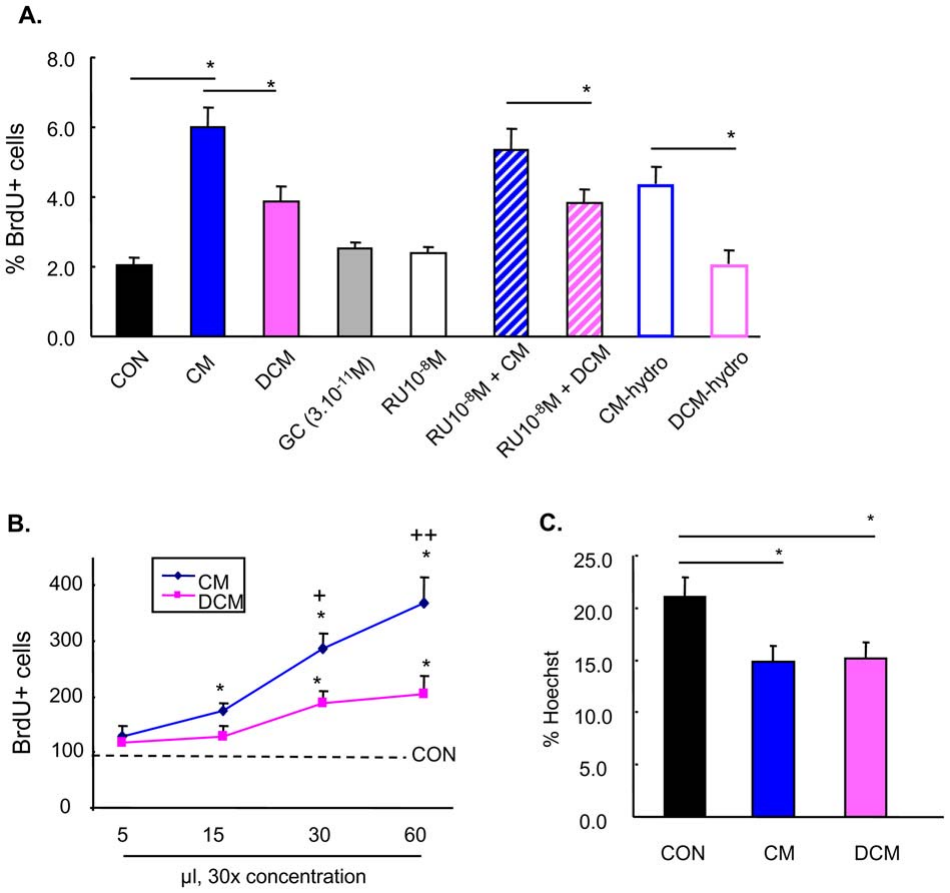
Conditioned medium from GC-treated astrocytes influences neurogenesis in hippocampal cultures. **A**, Treatment of primary hippocampal cultures with DCM (conditioned medium harvested from GC-treated astrocytes) attenuated the mitotic effects of CM (conditioned medium from normal astrocytes), measured by BrdU incorporation and Ki67 immunostaining. Conditioned media were prepared by either serial concentration (to reduce dexamethasone concentrations to <3.10^−11^ M; CM and DCM) or physical absorption of dexamethasone (CM-hydro and DCM-hydro). The effects of DCM could not be antagonized with GR antagonist, RU38486 (10^−8^ M). Neither dexamethasone (3.10^−11^ M) nor RU38486 (10^−8^ M) exerted significant effects on neural proliferation, and DCM-hydro attenuated the mitotic effects of CM-hydro to similar extents. **B**, CM caused a dose-dependent increase in neural proliferation, whereas DCM reduced the stimulatory effects of CM. **C**, Apoptosis in primary hippocampal cells in culture were reduced by CM and DCM to similar extents. Numerical data represent mean ± SD. * p<0.05 vs. CON, + p<0.05 vs. corresponding CM group.

Given the complexity of the above reported cytokine expression patterns, we next investigated whether GC treatment of astrocytes alters the expression of cytokines implicated in neural cell turnover. To this end, the effects of conditioned medium (CM) or conditioned medium from DEX-treated (DCM) astrocytic cultures (from which small [<MW 3 Kd] molecules were diluted out serially [final DEX levels: 3.10^−11^ M] or excluded by physical adsorption) on neurogenesis and apoptosis in primary hippocampal cultures was monitored. As shown in Fig. 8A, neural precursor cell proliferation was promoted by CM, an effect that was dose-dependently attenuated when astrocytes were treated with GC (DCM) (Fig. 8B). Neural precursor cell proliferation was not observed when cultures were exposed to DEX at a concentration of 3.10^−11^ M; this, together with the finding that the GR antagonist RU38486 failed to block the anti-proliferative effects of DCM, indicates that the effects of DCM did not result from the effects of residual GC in the CM. Lastly, both CM and DCM significantly, and to similar extents, attenuated apoptosis (Fig. 8C).

## Discussion

The pleiotropic roles of astrocytes have recently been extended to include the regulation of neurogenesis, migration and synaptic modulation [42], [43], with astrocytic loss and dysfunction being increasingly implicated in the pathogenesis of psychiatric disorders such as major depression [17], [19]–[21], [44]. However, while some authors reported a loss of astrocytes [16], [20], others reported an increase in astrocyte densities [45] in the hippocampus of depressed human subjects and animal models of depression. Accordingly, the present study involved a detailed analysis of the direct effects of GC on hippocampal astrocytic fate. In light of previous demonstrations that GC - strongly linked to depression - induce apoptosis in a subpopulation of hippocampal neurons [22]–[24], [26], [46], we specifically investigated whether GC influence astrocytic numbers through a similar process.

A first experiment in neonatal and adult rats revealed that, unlike hippocampal neurons, GFAP-labeled astrocytes do not succumb to apoptosis after GC administration. This finding was confirmed in more detailed analyses performed on primary hippocampal cultures (containing neurons and glia) as well as cultures enriched in hippocampus-derived astrocytes. Since the apoptotic process, from cell rounding and membrane blebbing through to lysis, can last between 12 and 24 h [47], and because individual cells may be asynchronous in their sensitivity, we here treated cultures with a synthetic GC (dexamethasone) for 48 h to capture all potential apoptotic events. Subsequently, early (stage I) or late (stage II) stages of DNA fragmentation were monitored, using immunoblotting with anti-phospho-H2A.X and pulsed-field gel electrophoresis (stage I) or TUNEL and Hoechst staining (stage II), respectively. Neither stage of DNA fragmentation was observed in astrocytes exposed to GC, indicating that astrocytes may be resistant to GC-induced apoptosis.

First attempts to identify the mechanisms that may contribute to the resistance of astrocytes to the apoptotic actions of GC revealed that, as compared to neurons, astrocytes have lower levels of reactive oxygen species (ROS) under resting conditions and generate lower ROS levels when exposed to GC. Thus, astrocytes are less likely to suffer from ROS-induced disruption of the mitochondrial membrane permeability, a major trigger of apoptosis [48]. Mitochondrial membrane permeability and thus, cell survival, is rheostatically regulated by pro- and anti-apoptotic members of the BCl2 family [24], [49]. Our finding that astrocytes can maintain a higher ratio of anti-apoptotic (BCl-2 and BCl-xL) to pro-apoptotic (Bax) protein levels after exposure to GC indicates that astrocytic refractoriness to GC-induced apoptosis may critically depend on this attribute.

Astrocytes exposed to GC display *moderate* increases in the levels of activated caspase 3, the so-called ‘executioner caspase’ even though they do not respond to this particular stimulus with signs of apoptosis. Our finding that staurosporine can trigger apoptosis in astrocytes not only verifies an intact apoptotic machinery but also indicates that, as compared to staurosporine, GC cannot elicit a sufficiently strong activated caspase 3 response. Previous studies have ascribed non-apoptotic functions to caspases (reviewed by Fernando and Megeney) [50]. For example, studies in cells of both, neuroepithelial [51]–[54] and mesodermal [55]–[57] origin suggest that activated caspase 3 plays a crucial role in cell differentiation. This notion is further supported by the recent finding that astroglial caspase 3 activation is not accompanied with cell death, but rather leads to cytoskeleton remodeling [58]. Our finding that GC cause astrocytic growth inhibition by inducing exit from the cell cycle (reduction of cyclin D1 and concomitant increase of p27) also supports the view that GC may contribute to the functional remodeling of astrocytes. Interestingly, in contrast to their targeting highly selective neuronal populations for apoptosis [59], GC have been reported to induce cell cycle arrest in a variety of neural cells, including neural precursors [60], microglia [61] and a neuroblastoma cell line [30]. To our knowledge, this is the first study to show that GC can also inhibit the proliferation of astrocytes while inducing their functional differentiation (see below). It therefore provides a new perspective on how elevated GC secretion may contribute to psychiatric illness.

Previous findings reported that GC alter the expression of astrocytic genes such as glutamine synthetase [62], GLT-1 [63] and interleukin-1 receptor [64]. These observations, together with results from the present study, show that the astrocytic transcriptome is influenced by GC. Interestingly and notwithstanding their potential roles in astrocytic insensitivity to GC-induced apoptosis, GC modulate the expression of a number of genes implicated in the regulation of neurogenesis in the hippocampus. However, the mRNA expression profiles of GC-treated astrocytes are difficult to interpret at present (e.g. the observed patterns of *bfgf* and *vegf* expression appear to be counter-intuitive). Importantly, this study shows that GC-induced changes in astrocytic function have a substantial influence over neurogenesis; the latter most likely involve the recruitment of, and cross-talk with, multiple effectors that play decisive roles in the pathways that regulate neural death and proliferation. To our knowledge, this represents the first report in which astrocytes are implicated as paracrine mediators of the negative effects of stress and GC on the proliferation of hippocampal neurons. To date, research on the regulation of neurogenesis and neuronal cell numbers by stress, GC and antidepressants [65] has been largely focused on the intrinsic mechanisms that control the birth and differentiation of neural precursors [60]. Our finding that neurogenesis is subject to extrinsic controls through GC-induced changes in astrocytic function adds a new dimension to present views of the pathophysiology of depression and other mental disorders in which dysregulation of hippocampal cytoarchitecture is causally implicated.

In summary, our studies show that hippocampal astrocytes do not enter the apoptotic pathway upon treatment with GC; accordingly, we conclude that the reduced number of astrocytes observed after exposure of animals to stress cannot be explained by GC-induced apoptosis. Our results indicate that, as compared to neurons, astrocytes are equipped with ROS load-reducing mechanisms that promote their survival. At the same time, GC appear to activate cellular pathways that result in an attenuation of neural proliferation. Together, these observations suggest that GC can dictate hippocampal architecture and ultimately function by initiating a hitherto undisclosed dialog between astrocytes and neurons.

## Materials and Methods

### Drugs and plasmids

The glucocorticoid receptor (GR) agonist dexamethasone (DEX), obtained from Merck (Darmstadt, Germany) in aqueous solution, was added to cultures for 48 h (24 h after transfection). The GR antagonist, mifepristone (RU38486; provided by the National Hormone and Pituitary Program, Torrance, CA) was added (10 µM) 1 h before the application of DEX. Cells undergoing mitosis were labeled by addition of 5-bromo-2′-deoxyuridine (BrdU; 20 µM; Sigma, St. Louis, MO) to cultures for 4 h. Staurosporine (Sigma) was used at 50 nM to induce apoptosis in astrocytes. The plasmids pBSII SK-Tα1-GFP (kind gift of Dr. Freda Miller) [27], [28] and pGFAP-GFP (kind gift of Dr. Helmut Kettenmann) [29] were used to label neurons and astrocytes, respectively.

### Primary hippocampal and enriched astrocyte cultures

Unless specified, all cell culture materials were purchased from Invitrogen (Eggenstein, Germany). Hippocampal neuronal cultures were prepared from Wistar rats aged 4 days (P4; Charles River, Sulzfeld, Germany), following a previously published protocol [66]. Transfections were carried out after 5–6 days in vitro (DIV), using Lipofectamine 2000 (Invitrogen) [66]. Transfection efficiency, judged by control transfection with pEGFP, was ∼10%.

Enriched astrocytic cultures were obtained from hippocampi from P4 rats [66], plated at a density of 130 cells/mm^2^ in DMEM containing 10% fetal bovine serum (FBS) and 1% kanamycin. After 12 days *in vitro* (DIV), cultures were shaken (260 rpm, 20 h) and washed with cold PBS. After discarding the supernatant, the residual cells were trypsinized and replated. Experiments were performed on astrocytes in their third passage *in vitro* and, depending on the specific treatments, were transferred into either DMEM/10% charcoal-stripped FBS (to exclude confounding by steroids in serum), DMEM/N2 Supplement or Neurobasal/B27 medium (to allow comparisons between glia and neurons).

### Conditioned medium

After washing with PBS, astrocytes were maintained for 48 h in Neurobasal A/B27 medium ± DEX. The growing medium (hereinafter referred to as conditioned medium, CM) was then harvested and centrifuged (300 rpm, 3 min, to remove residual cellular material); supernatants were then either concentrated or extracted to exclude DEX. For concentration, supernatants were run through Vivaspin columns (Vivaspin20, Sartorius, Aubagne, France) to concentrate peptides with an *Mr* >3 kD; smaller molecules, including DEX at an initial concentration of 10^−5^ M, were washed out serial dilution-concentration steps to reach a estimated final concentration of DEX that was <3.10^−11^ M. To extract DEX, supernatants were run through Speedisk H_2_O-Phobic DVB polymer columns (JT Baker, Phillipsburg, NJ). Complete removal of DEX from CM and DCM was evidenced by the disappearance of the phobic indicator, phenol red.

### Quantitative PCR

Total RNA was isolated (RNAeasy kit; Qiagen, Hilden, Germany) and reverse transcribed with Superscript II RNA H-reverse transcriptase (Invitrogen) and custom-synthesized Oligo-dT12-18 primers (MWG Biotech, Ebersberg, Germany). Quantitative PCR (qPCR) was performed with a LightCycler (Roche, Mannheim, Germany) in 10 µl mixtures containing 2 µl of 5X master mix (FastStart DNA SYBR green I; Roche), 5 µl of water, 0.5 µl of each primer and 2 µl of extracted DNA. The reaction was performed with preliminary denaturation for 10 min at 95°C (slope, 20°C/s), followed by 40 cycles of denaturation at 94°C (5 s), annealing (5 s) at 65°C and extension at 72°C (10 s). Relative mRNA expression ratios (housekeeping genes: *actin* and *gapdh*) were subsequently calculated. The following primers were used:

*bax* (174 bp) fwd: *CTGCAGAGGATGATTGCTGA*; rev: *GATCAGCTCGGGCACTTTAG*

*bcl-2* (251 bp) fwd: *CGGTGGTGGAGGAACTCTTC*; rev: *CAGCCAGGAGAAATCAAACAGA*

*bcl-2* (251 bp) fwd: *TGACCACCTAGAGCCTTGGAT*; rev: *CAGGAACCAGCGGTTGAAA*

*fas-l* (255 bp) *fwd: AAGGAGTGTGGCCCACTTAAC*; rev: *CTTCTCCTCCATTAGCACCAG*

*tnfα* (221 bp) *fwd: CCCAGACCCTCACACTCAGATCAT*; rev: *GCAGCCTTGTCCCTTGAAGAGAA*

*trail* (167 bp) fwd: *GCTTGCAGGTCAAGAGGCAAC*; rev: *TCTCCGAGTGATCCCGGTAATG*

*tweak* (154 bp) fwd: *CTGTCAGGTGCACTTTGATGAG*; rev: *AGCAAGTCCAGCTTCAGGTAGA*

*bdnf* (111 bp) fwd: *AAGGCTGCAGGGGCATAGAC*; rev: *TGAACCGCCAGCCAATTCTC*

*ngf* (142 bp) fwd: *CCAAGCACTGGAACTCATACTGC*; rev: *CTGCTGAGCACACACACGCAG*

*bfgf* (216 bp) fwd: *GACCCACACGTCAAACTACA*; rev: *TTTCAGTGCCACATACCAAC*

*vegf* (196 bp) fwd: *CCTGGTGGACATCTTCCAGGAGTACC*; rev: *GAAGCTCATCTCTCCTATGTGCTGGC*

*gapdh* (116 bp) fwd: *TGGAGAAACCTGCCAAGTATG*; rev: *GTTGAAGTCGCAGGAGACAAC*

*actin* (76 bp) fwd: *GGGAAATCGTGCGTGACATT*; rev: *GCGGCAGTGGCCATCTC*

### Pulse-field gel electrophoresis

Fragmentation of high molecular weight (HMW) DNA was assayed by pulse-field gel electrophoresis (CHEF-DR II; Biorad, Hercules, CA). Approximately 5.10^6^ astrocytes per condition were suspended in 40 µl of PBS, mixed with an equal volume of warm 1% Seakem gold agarose (SKG; Bio Whittaker Molecular Applications, Rockland, MD) in 0.5X TBE buffer (45 mM Tris, 45 mM boric acid, 1 mM EDTA, pH 8.3), and transferred to block molds (Biorad). Agarose blocks were incubated at 50°C in 1 ml of NDS buffer (1% laurylsarkosyl, 10 mM Tris, 0.5 M EDTA, pH 9.5) containing 200 µg/ml proteinase K, and then in NDS buffer containing 10 µg/ml RNase; each incubation lasted 24 h. The blocks were inserted into wells of a 1% SKG gel in 0.5X TBE, and electrophoresed at 6V/cm for 14 h at 14°C with a switch time of 5–50 s.

#### *Reactive oxygen species (ROS) generation*

Generation of ROS was assayed by allowing dihydroethidium (DHE; 5 µM) to react (30 min; 37°C) with cellular superoxide ions to yield a red fluorescent ethidium product. After washing and fixation in 4% PFA, ethidium accumulation in the cell nucleus was monitored by fluorescence microscopy (excitation, 520 nm; emission, 590 nm). Observations were made in hippocampal (mixed cell types and astrocyte-enriched) cultures under basal conditions and after exposure to DEX.

#### *Immunochemistry*

Cells were fixed in 4% paraformaldehyde, permeabilized in Triton-X100/PBS (0.3%) and blocked in 5% donkey serum/0.3% Triton (30 min) before incubation (4°C, overnight) with primary antibodies: anti-BrdU (1∶200; DAKO, Hamburg, Germany), anti-GFAP (1∶2000; DAKO), anti-GR (1∶300; Santa Cruz) and anti-active caspase 3 (1∶200; Cell Signaling/NEB, Frankfurt, Germany). For BrdU staining, cells were permeabilized, treated with 2*N* HCl (30 min) and incubated with anti-BrdU. After washing in PBS, cells were incubated (1 h, RT) with appropriate secondary AlexaFluor 488/594 conjugates (1∶500; Invitrogen). Nuclei were stained with Hoechst 33342 (1 µg/ml in PBS, 10 min). TUNEL histochemistry was performed as previously described [26], using FITC- or Texas Red-conjugated avidin (Vector Labs; Burlingame, CA) for signal visualization. Double staining of H2A.X and calbindin D28K or GFAP was performed using the following antisera (48 h incubations at 4°C): mouse anti-phospho-H2A.X (Millipore, Schwalbach, Germany; 1∶500) and rabbit anti-calbindin D28K (Millipore; 1∶500) or rabbit-anti-GFAP (DAKO). Appropriate Alexa conjugates were used to visualize immunoreactive signals. Specimens were examined on an Olympus BX-60 microscope, video-lined to a computer equipped with image-processing software (ImagePro, Media Cybernetics, Bethesda, MD). Cell counts were performed on 10 individual microscopic fields (0.072 mm^2^), randomly chosen across two diameters of each coverslip (400*X* magnification). An average of 1,000 cells or 100 transfected cells were sampled on each coverslip; results shown represent values from 6–9 coverslips/treatment.

#### Apoptosis in rat hippocampus

Experiments were conducted in accordance with local regulations (Regierung von Oberbayern License 2531-22-07) and European Union Directive (EU8869/10). Male Wistar rats born in-house to mothers from Charles River (Sulzfeld, Germany) were housed under standard laboratory conditions (12 hours light cycle; food and water available *ad libitum*). Rats (1 day or 3 months old) received daily s.c. injections of either vehicle (saline; n = 5) or a tapering dose of DEX (days 1–3: 200 µg/kg/d; days 4–7: 100 µg/kg/d; n = 6) and were sacrificed 24 h after the last injection. Brains were snap-frozen in a bath of isopentane and serial cryo-sections (20 µm each at intervals of 160 µm) were thaw-mounted onto gelatin-subbed glass slides, air-dried, and stored at −80°C until processing for immunohistochemistry. Incidence of apoptosis in calbindin D-positive neurons and GFAP-positive astrocytes was detected by phospho-H2A.X-staining. Sections were examined by confocal laser-scanning microscopy (Olympus IX81; 60X water-immersion lens) and results shown derive from evaluation of cells randomly selected within defined hippocampal subregions (100 neurons, 100 astrocytes; 4 sections per animal).

#### Lysate preparation and western blotting

Cells were lysed in 100 mM Tris-HCl, 250 mM NaCl, 1 mM EDTA, 5 mM MgCl2, 1% NP-40, a cocktail of protease inhibitors (Complete Protease Inhibitors; Roche, Mannheim, Germany) and phosphatase inhibitors (Sigma) for 30 min and cleared by centrifugation at 13,000 *g* for 20 min. After determination of protein concentration (Lowry method), samples were separated by SDS-PAGE on 10%-15% polyacrylamide gels, and transferred to nitrocellulose membranes. Membranes were blocked in PBS containing 5% non-fat milk and 0.2% Tween-20, and incubated overnight with antisera against phospho-H2A.X (1∶1000), active caspase 3 (1∶200), cyclinD1 (1∶300; Santa Cruz), cyclin E (1∶500; Santa Cruz), CDK6 (1∶500; Santa Cruz) or p27 (1∶500; Santa Cruz). Specific protein bands were revealed by enhanced chemiluminescence (GE Life Sciences, Freiburg, Germany), after incubation with appropriate horseradish peroxidase-IgG conjugates (GE Life Sciences).

#### *Statistics*

All numerical data (mean ± SEM) were subjected to ANOVA and appropriate post-hoc analysis, using SPSS software (v.10.0; SPSS Inc, Chicago, IL). The level of significance was preset at p<0.05.
